# The efficacy and safety of opening-wedge high tibial osteotomy in treating unicompartmental knee osteoarthritis: Protocol for a systematic review and meta-analysis

**DOI:** 10.1097/MD.0000000000014927

**Published:** 2019-03-22

**Authors:** Si-cong Huang, Yu-fang Chen, Xue-dong Liu, Yan-hong Han, Yi-qun Li

**Affiliations:** a The Second People's Hospital of Foshan (Affiliated Foshan Hospital of Southern Medical University), Foshan; b The Second Clinical College of Guangzhou University of Chinese Medicine, Guangzhou, China.

**Keywords:** closing-wedge high tibial osteotomy, knee, meta-analysis, opening-wedge high tibial osteotomy, osteoarthritis, randomized control trials

## Abstract

**Background:**

High tibial osteotomy (HTO) is an effective surgical technique that can stop or inhibit the progression of unicompartmental knee osteoarthritis (KOA) to avoid or postpone the need for knee arthroplasty in patients. Whether opening-wedge high tibial osteotomy (OWHTO) is superior to closing-wedge high tibial osteotomy (CWHTO) in treating unicompartmental KOA remains controversial.

**Methods:**

Databases (Cochrane Library, EMBASE, and PubMed) were searched from their establishment to July 1, 2018 for randomized controlled trials comparing the application of OWHTO to CWHTO in patients with unicompartmental KOA. The methodological quality of each included study was assessed according to the Cochrane Handbook for Systematic Reviews of Interventions guideline. Review Manager 5.3.5 software (Cochrane Collaboration, Oxford, UK) was used to synthesize the final results.

**Results:**

The results will provide useful information about the effectiveness and safety of OWHTO in patients with unicompartmental KOA.

**Conclusion:**

The findings of the study will be published in a peer-reviewed journal.

**PROSPERO registration number:**

CRD4201811805.

## 1 Introduction

Osteoarthritis (OA) of the knee is one of the most common joint disorders, and it may lead to joint dysfunction, that is, a reduction of joint motion and physical disability, as a result of tissue degeneration and destruction and the loss of articular cartilage.^[1,2]^ High tibial osteotomy (HTO) is performed to stop or inhibit the progression of unicompartmental knee osteoarthritis (KOA) and to avoid or postpone knee arthroplasty in patients with unicompartmental KOA.^[3–7]^ Among them, opening-wedge high tibial osteotomy (OWHTO) and closing-wedge high tibial osteotomy (CWHTO), which involve stabilization with a locking plate, are 2 of the most frequently used techniques.^[8–10]^ OWHTO is a relatively new technology. Compared with CWHTO, there is less involvement in the surgical technique, such as a single patella incision without the need for humeral osteotomy.^[6]^ However, there are ongoing discussions regarding choosing the of method for preoperative planning, the operative technique, and the osteotomy site.^[11]^ Alterations in joint line angles, the posterior tibial slope, the patellar height (PH), correction accuracy, and OWHTO and CWHTO survivorship durations are among the controversial issues.^[11]^ To help resolve these existing uncertainties, we performed a meta-analysis to evaluate the differences in clinical outcomes between patients undergoing OWHTO and CWHTO and to explore whether OWHTO is superior to CWHTO.

## 2 Methods

This study will be conducted in accordance with the Preferred Reporting Items for Systematic Reviews and Meta-analysis (PRISMA) Statement and is registered in PROSPERO (registration number: CRD4201811805). The study was approved by the ethics committee of The Second People's Hospital of Foshan.

### 2.1 Eligibility criteria

#### 2.1.1 Type of study

Randomized controlled trials (RCTs) that compared radiographic and/or clinical outcomes between OWHTO and CWHTO will be included. Only articles published in English will be included.

#### 2.1.2 Type of patients

All participants will be diagnosed with unicompartmental KOA. The patient's gender, age, and grades of unicompartmental KOA will not be limited.

#### 2.1.3 Type of interventions

The intervention in eligible researches of interest is the experimental group, which will be treated with OWHTO, while the control group will receive the CWHTO.

#### 2.1.4 Type of outcomes

The primary outcomes were Hospital for Special Surgery knee scores and visual analog scale knee pain scores. The secondary outcomes were posterior tibial slope angle, PH, hip-knee-ankle angle, walking distances, complications, and survivorship durations.

### 2.2 Search strategy

This study search will be mainly based on electronic databases, including Cochrane Library, EMBASE, and PubMed databases. The title, abstract, and MeSH search terms included (“Open”) and (“Closed” or “Closing”) and (“Osteotomy” or “Tibial”). Studies published in English before July 1, 2018 were considered. Related references in the identified studies and previous systematic reviews for other potentially relevant literature were manually searched. After the initial electronic search, the remaining studies are reviewed according to the eligibility criteria.

### 2.3 Study selection and data extraction

**Figure 1:**
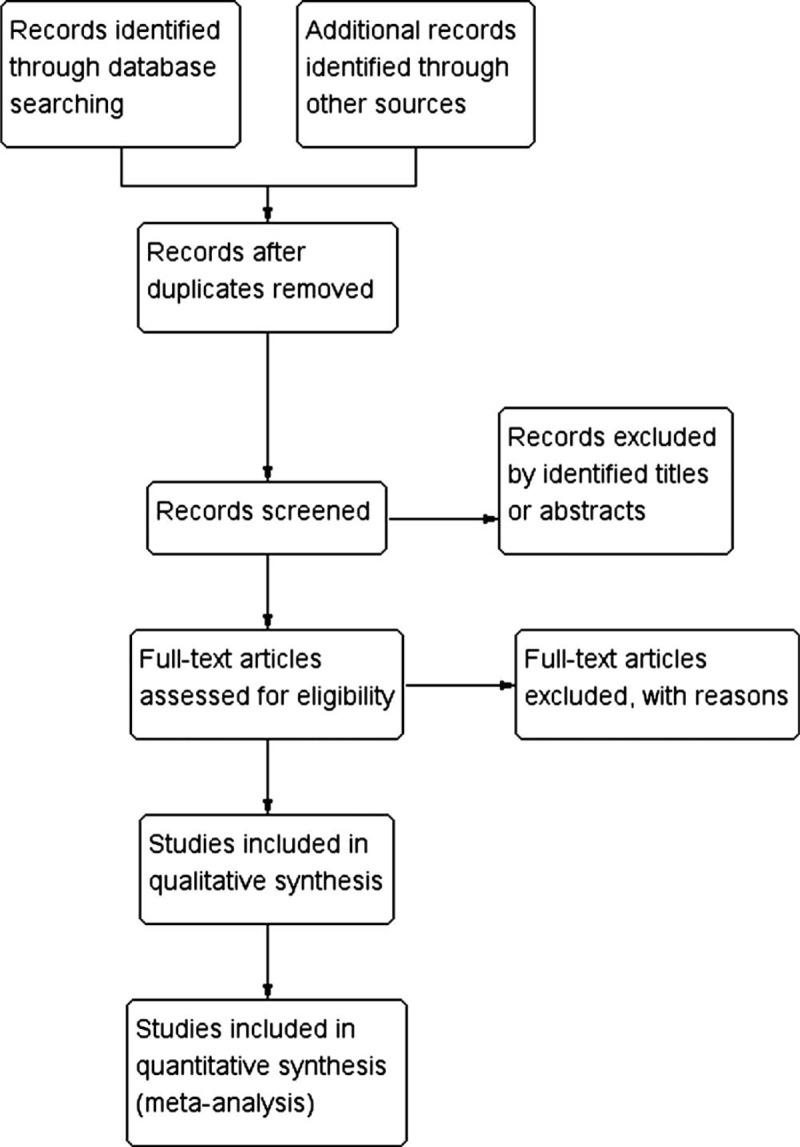
Flow diagram of study selection process.

Two reviewers independently performed study selection and data extraction. The selection process will be presented in Figure 1. Data extraction will include general data (first author, publication year, and study design), population characteristics, follow-up assessment method, details of the OWHTO and CWHTO group, as well as relevant details of radiographic outcomes and clinical outcomes.

### 2.4 Quality assessment

The methodological quality of each included study was independently assessed by 2 reviewers according to the Cochrane Handbook for Systematic Reviews of Interventions guideline. Two reviewers evaluated each of the following domains: random sequence generation and allocation concealment, blinding of participants and personnel, blinding of outcome assessments, incomplete outcome data, selective reporting, and other sources of bias. Any disagreements were arbitrated by the corresponding author.

### 2.5 Statistical analysis

Review Manager 5.3.5 (Cochrane Collaboration, Oxford, UK) was used to calculate the effect sizes of the included studies. For dichotomous variables, the odds ratio and 95% confidence interval (CI) were derived for each outcome; for continuous variables, we calculated the weighted mean difference and 95% CI. The statistical heterogeneity was calculated using the chi-squared test and *I*^2^ statistic. Pooled analyses were performed with the application of a fixed effects model in case of no significant statistical heterogeneity; otherwise, a random effects model was applied. For all analyses, *P* < .05 was considered statistically significant.

## 3 Discussion

OA can affect all 3 compartments of the knee. However, approximately one-third of patients are afflicted in only 1 of the compartments, and many of them have unicompartmental OA.^[12]^ The purpose of surgery for unicompartmental KOA is to reduce pain, restore joint function, and improve the patient's future quality of life.^[13]^ Patients undergoing HTO can benefit from natural joint preservation, with almost no effects on physical loading.^[13]^ Some previous systematic reviews and meta-analysis^[2,14,15]^ have shown that OWHTO is an effective treatment in unicompartmental KOA patients. However, the previous conclusions were reached on the basis of the observational study and the small number of RCTs, and thus, the effectiveness of OWHTO in the treatment of unicompartmental KOA still lacks high-level evidence support. Recently, some potential clinical studies on this topic have been published. In order to further determine the comparative role between OWHTO and CWHTO in unicompartmental KOA patients, we designed this updated systematic review and meta-analysis for the purpose of providing useful information about the effectiveness and safety of OWHTO for patients with unicompartmental KOA. The findings of the study will be published in a peer-reviewed journal.

## Acknowledgments

We thank American Journal Experts for editing the English text of a draft of this manuscript.

## Author contributions

**Conceptualization:** Si-cong Huang, Yi-qun Li.

**Data curation:** Si-cong Huang, Yu-fang Chen, Yan-hong Han.

**Formal analysis:** Si-cong Huang, Yan-hong Han.

**Funding acquisition:** Yi-qun Li.

**Investigation:** Si-cong Huang, Yu-fang Chen, Xue-dong Liu, Yan-hong Han, Yi-qun Li.

**Methodology:** Yu-fang Chen, Xue-dong Liu.

**Project administration:** Xue-dong Liu, Yan-hong Han.

**Resources:** Xue-dong Liu.

**Software:** Si-cong Huang, Yu-fang Chen, Yan-hong Han.

**Visualization:** Xue-dong Liu.

**Writing – original draft:** Si-cong Huang, Yu-fang Chen.

**Writing – review & editing:** Si-cong Huang, Yiqun Li.
